# Evaluation of Mini-Preauricular Incision in the Surgical Management of Condylar Fracture

**DOI:** 10.7759/cureus.31725

**Published:** 2022-11-21

**Authors:** Praveen Kumar, Balaji Jeyaraman, Davidson Rajiah, Arunkumar Kamalakaran, Rohini Thirunavukkarasu, Triveni Palani

**Affiliations:** 1 Oral and Maxillofacial Surgery, Tamil Nadu Government Dental College and Hospital, Chennai, IND

**Keywords:** mini-preauricular incision, mandibular fracture, open reduction internal fixation, condylar fracture, modified preauricular incision

## Abstract

Introduction

Mandibular fractures have the highest incidence next to nasal bone fractures of which condylar fractures account for one-third of it. Various approaches for condylar fracture include intraoral and extraoral approaches such as coronal, preauricular, postauricular, endaural, endoscopic, rhytidectomy, transparotid, submandibular, and retromandibular approaches. The purpose of this study was to evaluate the mini-preauricular incision in open reduction and internal fixation of condylar and subcondylar fractures of the mandible.

Materials and methods

Twenty patients with condylar fracture underwent open reduction and internal fixation under general anesthesia using a modified mini-preauricular incision and subdermal dissection approach. Parameters assessed were pain, mouth opening, occlusal derangement, accessibility of fracture site, duration of surgery, neurosensory deficit (facial nerve), postoperative edema, wound infection, wound dehiscence, and scar. Patients were followed up at an interval of one week, one month, three months, and six months.

Results

On comparing the parameters preoperatively and postoperatively, occlusal derangement, mouth opening, and pain showed statistical significance with a p-value of 0.01, while nerve weakness and scar assessment showed a high level of statistical significance with a p-value of 0.001. The anatomical reduction of the condyle and internal fixation with miniplates was easy when this approach was used. Patients showed transient facial nerve paralysis only. No permanent damage was noted. The resultant scar was aesthetically acceptable.

Discussion

The mini-preauricular approach is an effective and safe technique for open reduction and internal fixation of condylar and subcondylar fractures. This approach provided good access, good cosmetic results, and patient satisfaction. This approach resulted in very less morbidity to the facial nerve.

## Introduction

Mandibular fractures have the highest incidence next to nasal bone fractures of which condylar fractures account for 30%-37% [1]. The decision to treat adult condylar fractures through closed or open techniques is a topic of controversy and debate in maxillofacial trauma. Current literature aims at achieving early functional rehabilitation through the benefits of open reduction with internal fixation. The two types of surgical approaches for the mandibular condyle are intraoral and extraoral. The extraoral incisions used to expose the condyle are as follows: coronal, preauricular, postauricular, endaural, endoscopic, rhytidectomy, transparotid, submandibular, and retromandibular approaches, of which the preauricular incision is the most common [2]. A preauricular incision is made anterior to the tragus along the natural crease. But the major problem encountered in this approach is that the lower portion of the condylar process is not much accessible, and rigid fixation of the same is comparatively difficult [3]. In addition to the above-mentioned drawbacks, the probability of facial nerve damage is also quite high in this incision.

Most recently, condylar fractures are being addressed using endoscopic equipment intraorally or extraorally with great success [4]. In order to avoid some of the shortcomings of earlier extraoral and endoscopic techniques for the repair of condylar and subcondylar fractures, a preauricular, mini-incision, open technique (PMIOT) was advocated as a new and reliable extraoral approach [5]. When compared to the conventional retromandibular approach where a 3-3.5 cm incision is placed below the earlobe, the PMIOT in spite of the lesser length of approximately 1.5-2 cm provides good surgical access. This study was previously presented as a thesis submission. The study was done to evaluate the mini-preauricular incision open technique for open reduction and internal fixation of condylar and subcondylar fractures of the mandible. The study was aimed at evaluating the effectiveness of modified preauricular mini-incision in the surgical management of condylar fractures.

## Materials and methods

**Figure 1:**
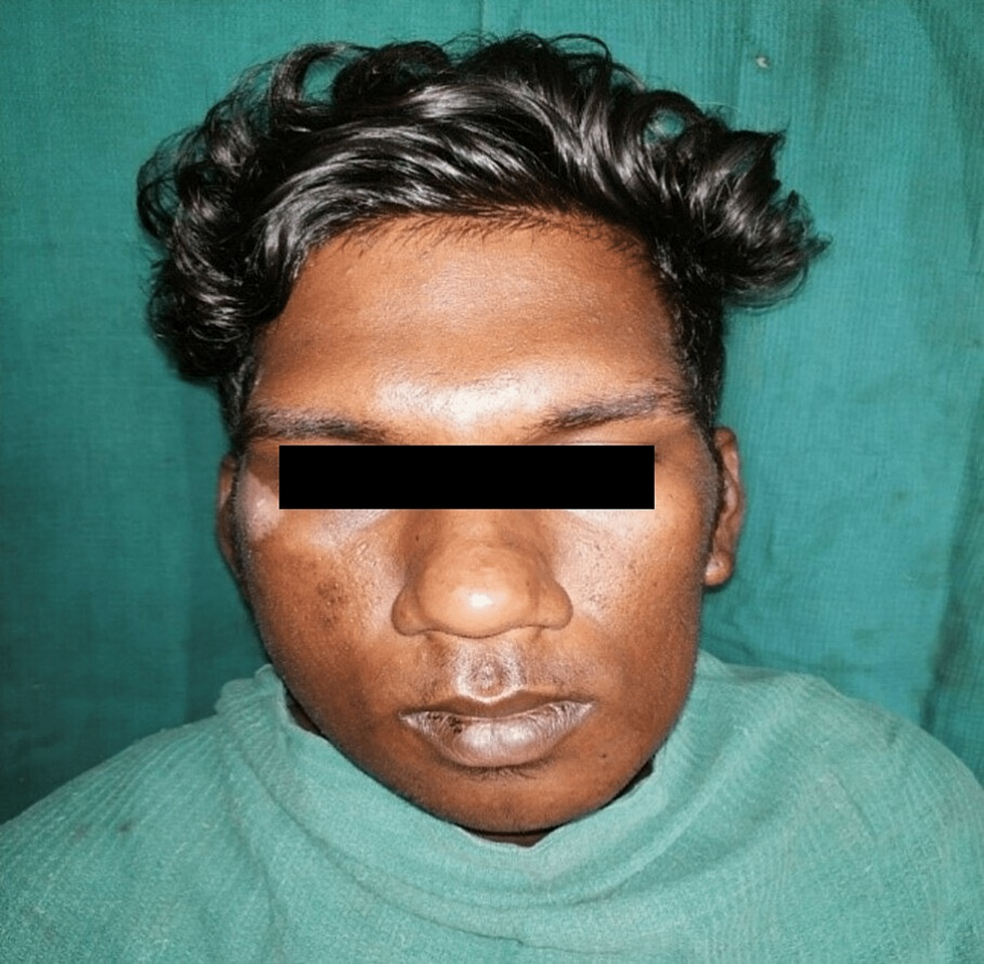
Preoperative clinical photograph

**Figure 2:**
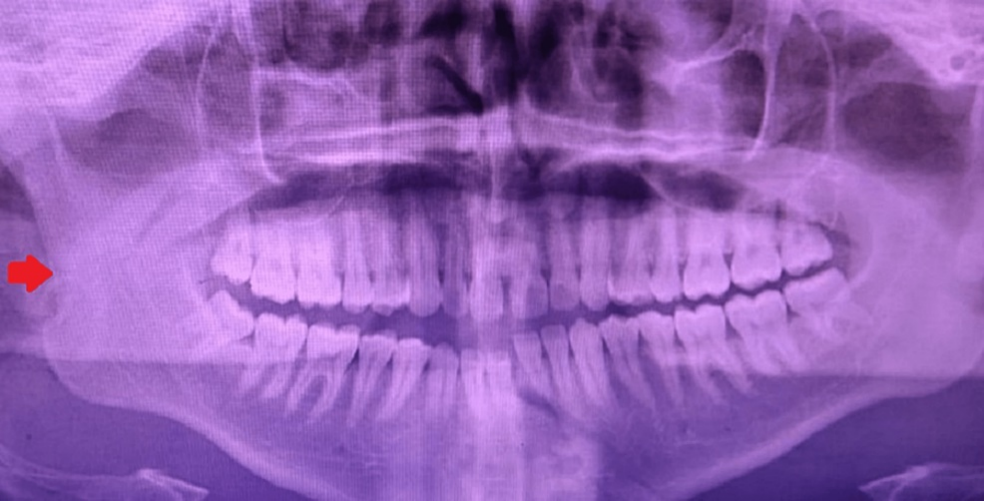
Preoperative orthopantamogram indicating fracture of the right subcondyle and left parasymphysis

The following study was performed after obtaining approval from the Institutional Review Board of Tamil Nadu Government Dental College and Hospital (Reference no.: 4/IRB/2018). A total of 20 patients were arbitrarily included in this study. Complete intraoral and extraoral examination, medical history, orthopantamogram, computed tomography scan of facial bones, and clinical photographs were obtained from the patients (Figures 1, 2). Patients with condylar fractures (Spiessl and Schroll types II, III, and IV) with or without other associated mandible fractures, patients in the age range of 16-60 years, healthy individuals with no debilitating systemic diseases, and patients consenting for surgery were included in this study. Patients with comminuted condyle fractures, pediatric condylar fractures, edentulous mandibular fractures, debilitating systemic diseases, and known drug allergies were excluded from the study.

**Figure 3:**
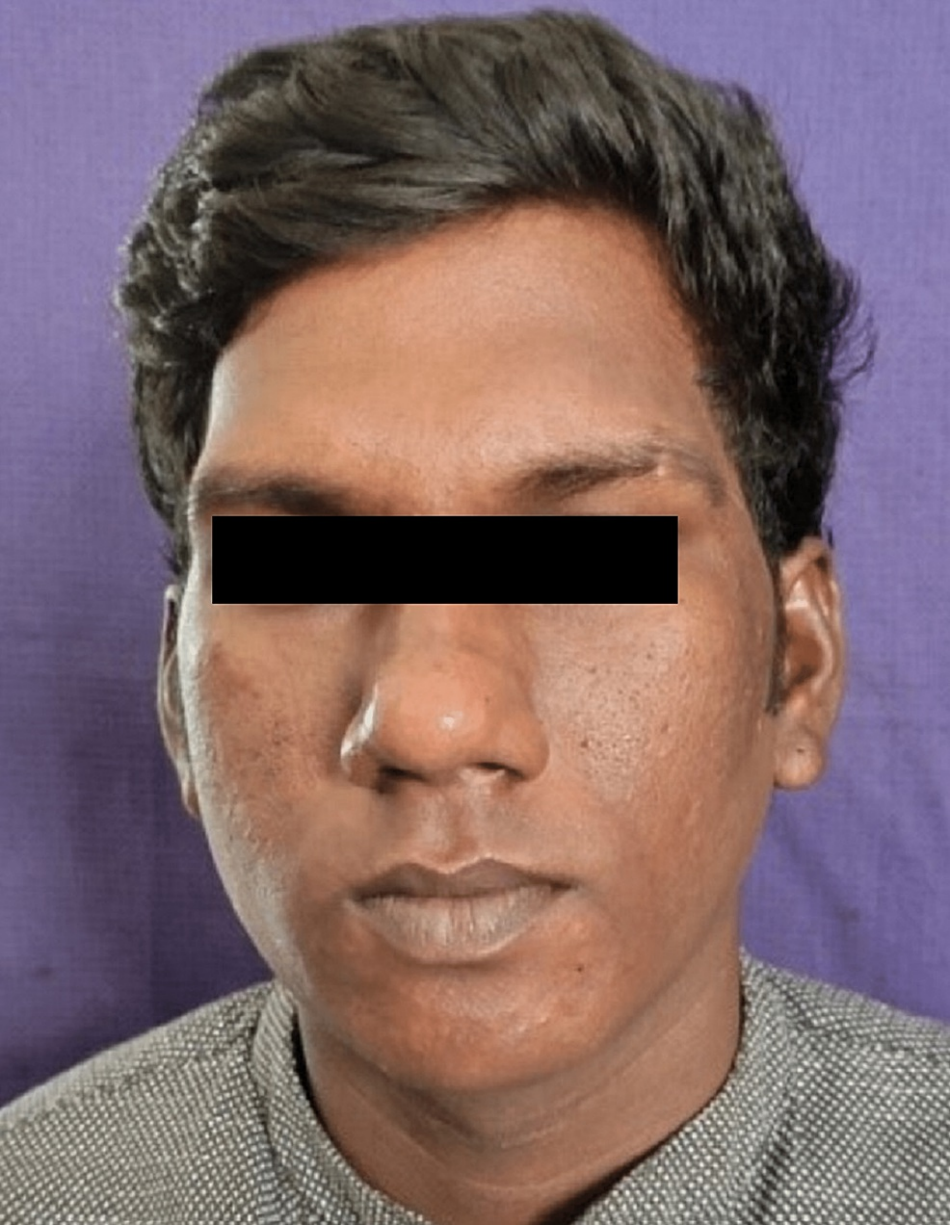
Postoperative clinical photograph

**Figure 4:**
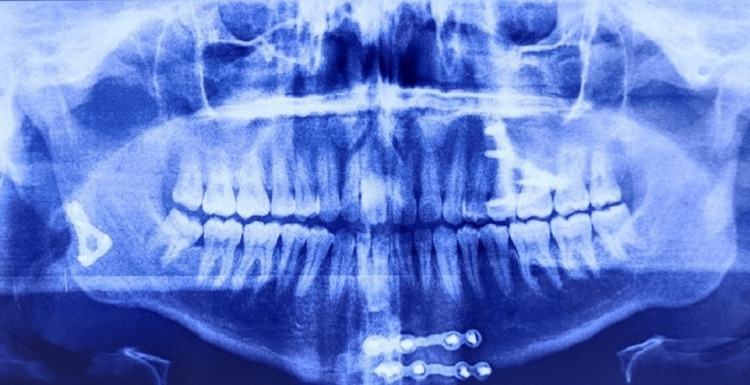
Postoperative orthopantamogram displaying miniplates in the right subcondyle and left parasymphysis regions

All patients underwent open reduction and internal fixation under general anesthesia using a modified preauricular mini-incision subdermal dissection approach. Internal fixation was done using a single delta or trapezoid mini-plate and 2 mm × 6 mm titanium screws. Patients were followed up at an interval of one week, one month, three months, and six months (Figures 3, 4). The parameters evaluated were pain, mouth opening, occlusal derangement, accessibility of fracture site, duration of surgery, neurosensory deficit (facial nerve), postoperative edema, wound infection, wound dehiscence, and scar formation.

Surgical procedure

**Figure 5:**
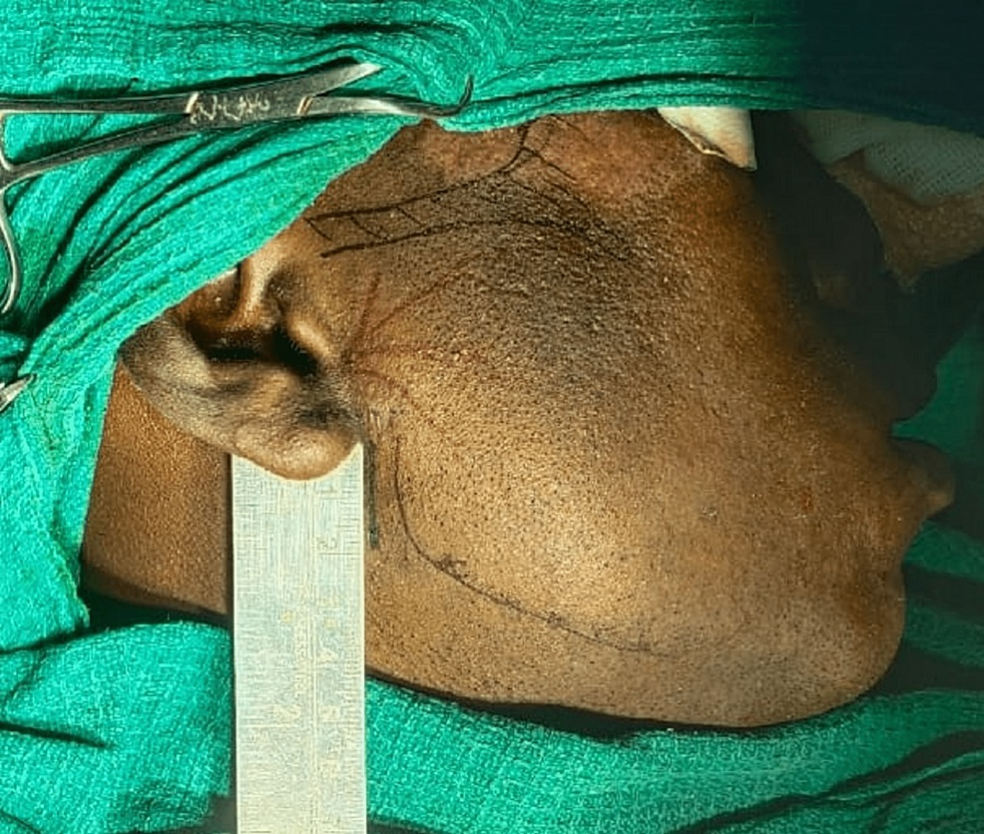
Mini-preauricular incision marked from the region below the earlobe to the angle of the mandible

**Figure 6:**
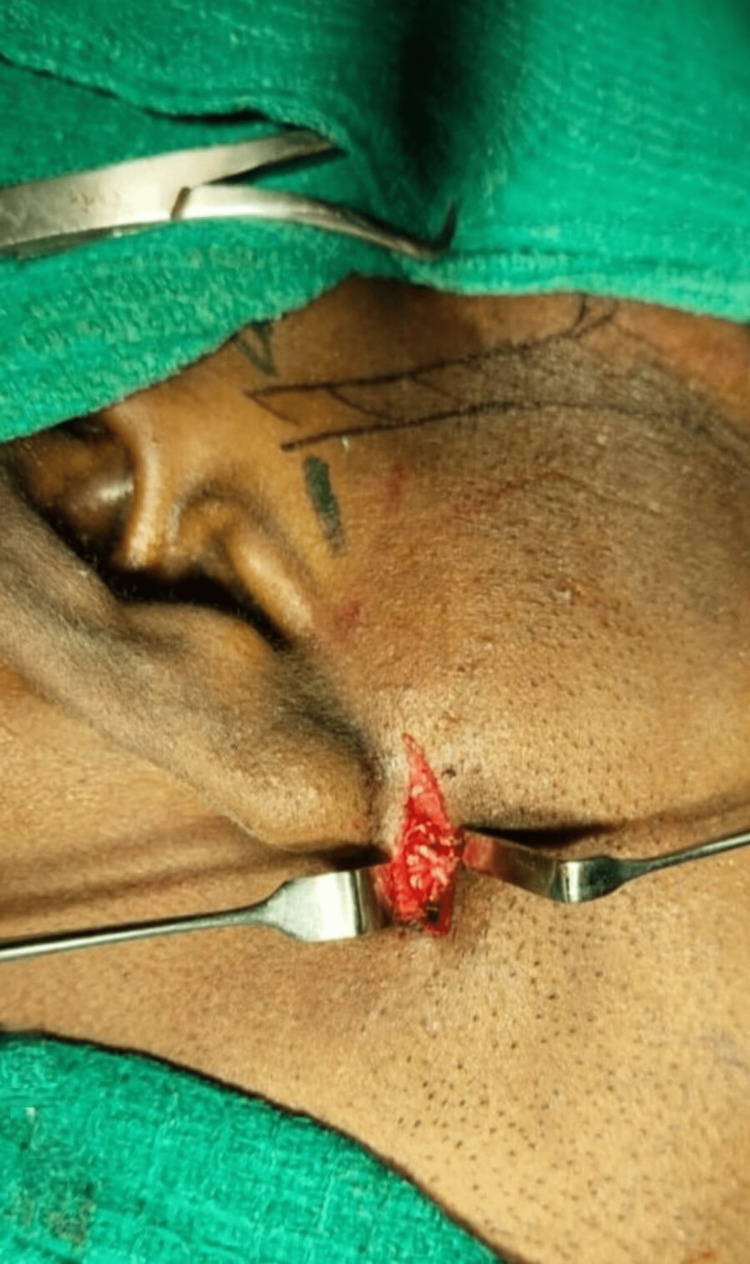
Incision placed and layerwise dissection done through the skin and subcutaneous tissue

After the clinical and radiographic assessment, the selected patients were planned for open reduction with internal fixation under general anesthesia. Oral prophylaxis was done followed by the placement of Erich’s arch bars, and occlusion was achieved by intermaxillary fixation with elastics whenever possible. Anatomical landmarks of the zygomatic arch, facial nerve along with its branches, and mandible were marked (Figure 5). Under general anesthesia, patients are painted and draped. A 2-cm long preauricular incision was drawn extending from below the center of the lower aspect of the ear lobe up to the angle of the mandible (Figure 6). In order to achieve a facial plane of dissection and vasoconstriction, 2% lignocaine with 1:100,000 adrenaline was injected into the marked incision site. A 2-cm incision was placed using a No. 15 surgical blade in the preauricular region following the marking.

Skin and subcutaneous layers were dissected to reach the angle of the mandible. Using the periosteal elevator attachments, the masseter muscle was severed followed by subperiosteal dissection until the subcondylar region was exposed. The facial nerve and parotid duct were preserved. A condylar fracture site was identified. To improve the access, complete muscle relaxation was provided by the anesthetist so as to reduce muscle contraction and ease fracture reduction. This led to an easier and more rigid fixation of fractures using delta or trapezoid plates along with 2 mm × 6 mm screws. The muscle layer was closed with simple interrupted sutures using 3-0 vicryl, while the skin was closed with subcuticular sutures using 5-0 prolene.

## Results

All 20 patients were taken up for open reduction and internal fixation under general anesthesia for a period of seven days following trauma. In all 20 patients, a mini-preauricular incision with a subdermal dissection approach was used along with the use of a paragingival incision as and when necessary (for the management of parasymphyseal fracture). In all 20 patients, titanium condylar plates (delta plate, trapezoid plate) were used for the fixation of subcondylar fractures, and 2 x 4 hole titanium miniplates were used for treating parasymphyseal fractures. There were no dropouts. All the patients were followed up at regular intervals of time. Preoperatively, radiological evaluation was done using orthopantamogram and computed tomography scans to locate the level at which the subcondyle was fractured and the deviation of the fractured condyle in degree, which was used as guidelines to evaluate the fracture reduction intra- and postoperatively. All the patients were followed up at intervals of one week, one month, three months, and six months postoperatively.

A visual analog scale was used to measure the pain; 0%-10.20% of the patients experienced moderate pain during the first postoperative week. All patients showed pain-free movements six months postoperatively. All patients had a mouth opening of less than 25 mm preoperatively, and this increased to 30-39 mm during the first postoperative week in 90% of patients. All patients achieved more than 40 mm mouth opening at the end of the first postoperative month. During the first postoperative week, 5% of the patients had mild occlusal derangement. Intermaxillary elastics were used for all the patients for a period of 10 days postoperatively, and the desired occlusion was achieved by the end of one postoperative month (Table 1).

**Table 1:** Pre- and postoperative assessment of pain, occlusal derangement, mouth opening, nerve weakness, and scar assessment N: Number of patients. * indicates statistical significance. ** indicates a high level of statistical significance.

Assessment	Preoperative		1 week		1 month		3 months		6 months		P-value
	N	%	N	%	N	%	N	%	N	%	
Pain											
0 (No pain)	0	0	0	0	2	10	20	100	20	100	0.01*
1 (Moderate pain)	0	0	4	20	18	90	0	0	0	0	
2 (Severe pain)	20	100	16	80	1	20	0	0	0	0	
Occlusal derangement											
1 (Present)	20	100	1	5	0	0	0	0	0	0	0.01*
2 (Absent)	0	0	19	95	20	100	20	100	20	100	
Mouth opening											
0 (˂25 mm)	20	100	0	0	0	0	0	0	0	0	0.01*
1 (30-39 mm)	0	0	18	90	0	0	0	0	0	0	
2 (˃40 mm)	0	0	2	10	20	100	20	100	20	100	
Nerve weakness											
1 (Present)	0	0	3	15	3	15	0	0	0	0	0.001**
2 (Absent)	20	100	17	85	17	85	20	100	20	100	
Scar assessment											
1 (Hypertrophic)			0	0	0	0	0	0	0	0	0.001**
2 (Conspicuous)			20	100	4	20	0	0	0	0	
3 (Inconspicuous)			0	0	16	80	20	100	20	100	

The time taken from the start of the incision to the closure of the wound was measured in minutes. The average operative time was 58.6 minutes, with a standard deviation of 5.3 minutes (Table 2). Subjective evaluation of surgical accessibility was done by the operating surgeon. There was good access in four patients as the condyle was more displaced. For the other 16 patients, the accessibility was excellent. Accessibility was measured based on the feasibility of reducing fractured segments and placement of miniplates and screws. Sixteen of the 20 patients had inconspicuous scars in the first postoperative week, wherein the scars are not noticeable after a month. Marginal mandibular nerve weakness was observed in three patients. The nerve weakness was present for a period of two months.

**Table 2:** Assessment of surgical time, surgical accessibility, postoperative edema, infection, and wound dehiscence N: Number of cases.

Surgery time in minutes	Mean ± SD
	58.6 ± 5.3
Surgical accessibility	N (%)
0 (Fair)	0(0)
1 (Good)	4 (20)
2 (Excellent)	16 (80)
Postoperative edema	N (%)
Present	1 (5)
Absent	19 (95)
Postoperative infection	N (%)
Present	0 (0)
Absent	20 (100)
Wound dehiscence	N (%)
Present	0 (0)
Absent	20 (100)

**Figure 7:**
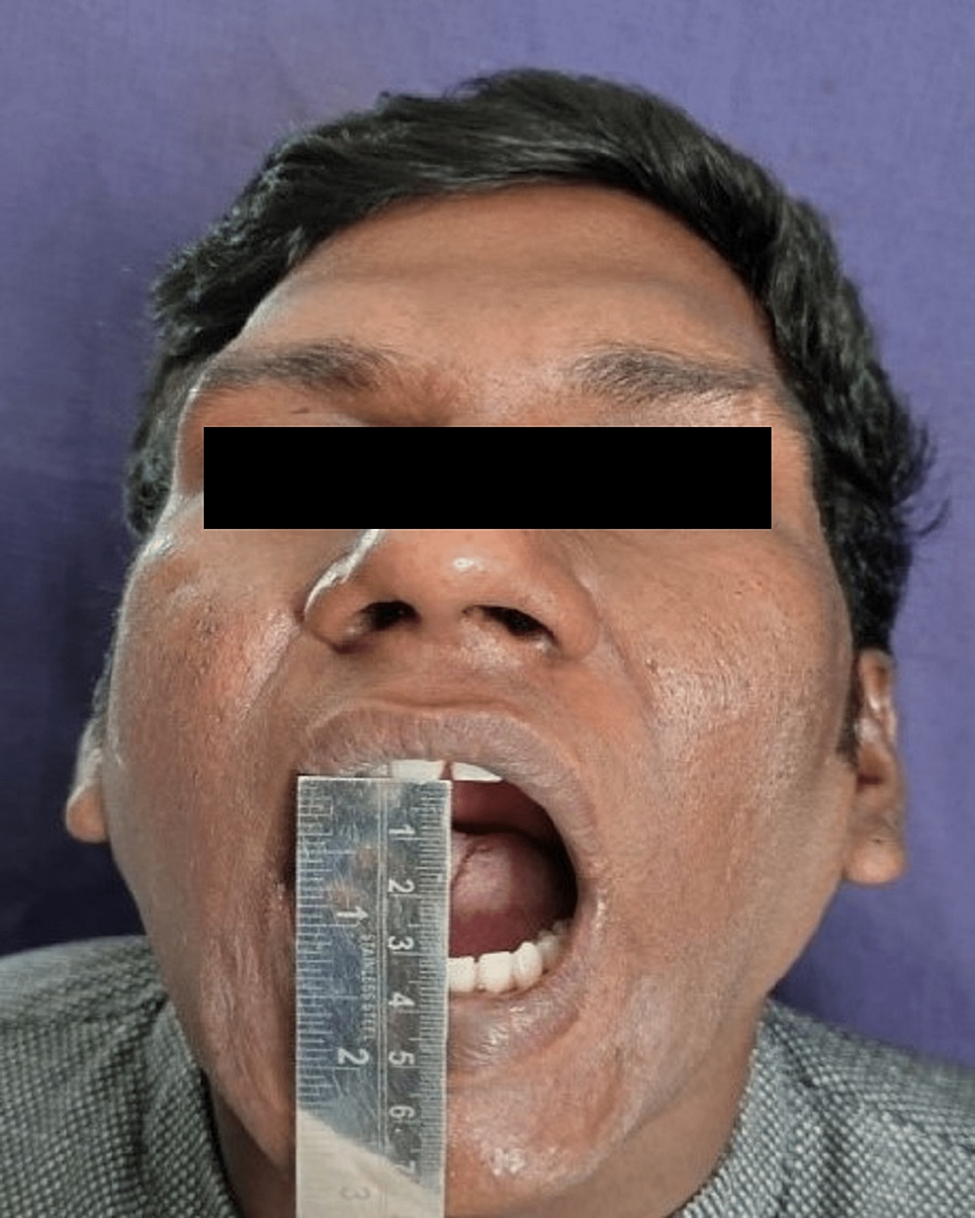
Postoperative mouth opening

**Figure 8:**
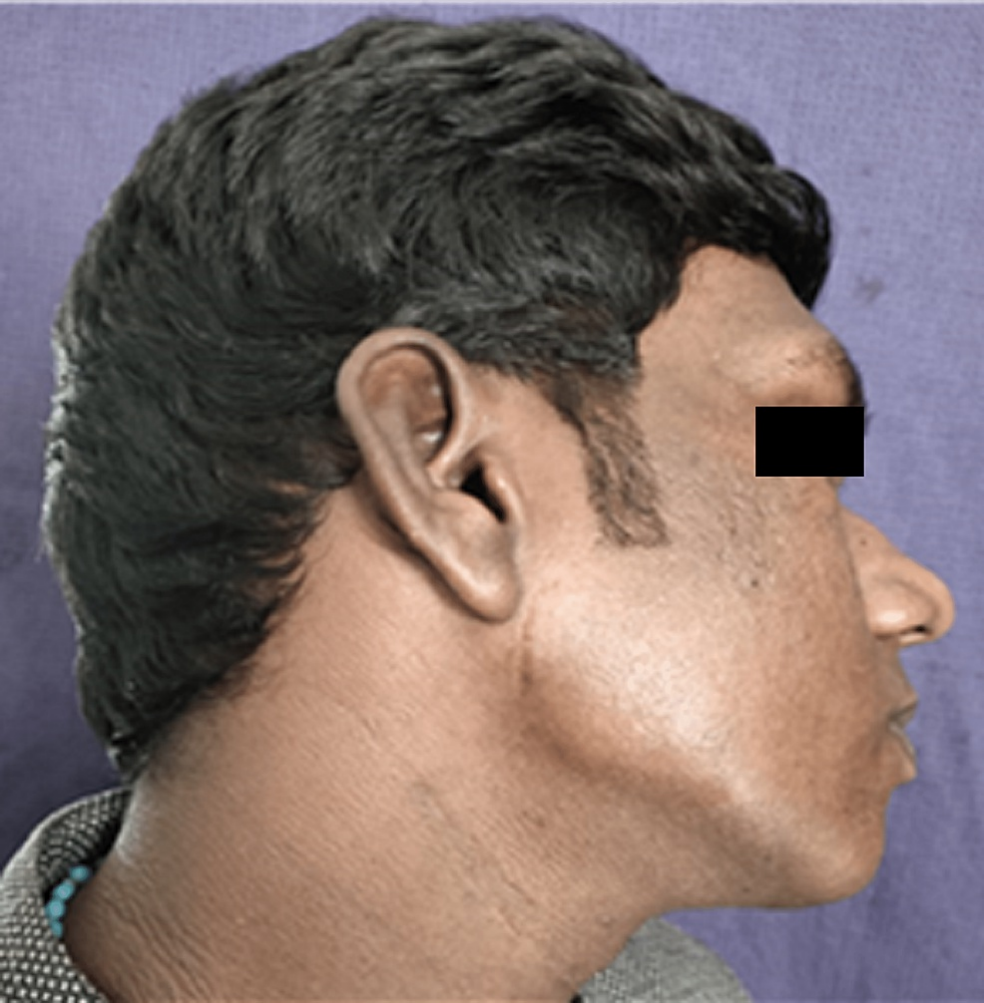
Postoperative scar

No permanent facial nerve disturbance was present at the end of six months (Figures 7, 8). On comparing the parameters preoperatively and postoperatively, occlusal derangement, mouth opening, and pain showed statistical significance with a p-value of 0.01, while nerve weakness and scar assessment showed a high level of statistical significance with a p-value of 0.001.

## Discussion

Endoscopy-assisted surgical management of condylar fracture also leaves a 4-5 cm length scar [6-8]. In spite of the lesser length of approximately 1.5-2 cm, surgical accessibility with this approach was found to be good (in 20% of patients) to excellent (in 80% of patients). An appropriate reduction was accomplished by the use of small retractors to retract the surrounding structures and the use of hooks, periosteal elevators, and condylar retractors to manipulate the fracture segment. Even when the incision was small, we were able to place trapezoidal plates and delta plates for stable fixation of subcondylar fractures along Champy’s line of osteosynthesis [9,10]. In addition, the surgical time was significantly decreased (mean: 58.6 minutes with a standard deviation of 5.3 minutes) due to the minimal incision, which in turn prevented the need for more widespread dissection during the condylar fracture reduction. Since open reduction and internal fixation were carried out, there was no requirement for any period of rigid postoperative intermaxillary fixation, which tends to be an added advantage. During the first postoperative week, 5% of the patients had mild occlusal discrepancies. In these patients, intermaxillary elastics were placed in the immediate postoperative period for 10 days, and satisfactory occlusion was achieved by the end of the postoperative month. With this incision, the postoperative mean mouth opening achieved was 41.4 mm, which was statistically significant when compared to the preoperative mouth opening. The preoperative occlusal discrepancy was managed by this approach. One of the most important advantages of this mini-preauricular incision was the inconspicuous postoperative scar when compared to other extraoral approaches and the excellent accessibility to the subcondylar fractures. Any type of mini-plate osteosynthesis can be achieved successfully.

Facial nerve paralysis is one of the most common complications in open reduction of the condylar fracture. There was no facial nerve damage in the high submandibular approach for a subcondylar fracture. Endoscopy-assisted repair of subcondylar fracture reported that 33% of patients had facial nerve paralysis, which resolved within three months [8,11,12]. In our study, facial nerve paralysis was encountered in three patients, and it was transient in nature and recovered completely after three months of follow-up. The incidence of facial nerve paralysis was very low (15% in the first postoperative week) and transient in nature, providing good cosmetic results [11,12]. In comparison to the other conventional approaches, modified preauricular mini-incision has the potential to leave inconspicuous scars without any damage to any essential anatomical structures such as the facial nerve, external auditory canal, parotid gland, and maxillary artery [5].

The length of incision to the fracture site is minimal (1.5-2 cm), and it also provides excellent exposure to the condylar fracture site. The anatomical reduction of the condyle and internal fixation with miniplates was easy when this approach was used. However, some difficulty was found in reducing the medially displaced condylar fragment. Facial nerve branches may be encountered in some of the patients, which can be directly visualized and safely retracted superiorly or inferiorly. The buccal branch of the facial nerve was most frequently encountered. In this study, patients were reported to have had facial nerve paralysis, which was transient in nature, and the recovery of function was earlier when compared to other approaches like the retromandibular and submandibular approaches. No permanent damage was noted. There was no statistical difference between preoperative and postoperative facial nerve function. This signifies that the mini-preauricular approach for open reduction and internal fixation of subcondylar fractures likely carries less risk of facial nerve damage compared to the traditional approaches. The resultant scar was acceptable to the patient.

The limitations of the study include a small sample size and a shorter duration of follow-up; however, long-term studies with larger sample sizes and comparative studies with other surgical approaches to condylar fractures are required.

## Conclusions

The mini-preauricular approach provides excellent exposure to the subcondylar fractures with a minimal incision length of 1.5-2 cm, which is less likely to injure the branches of the facial nerve when compared to the conventional retromandibular approaches. The resultant scar was acceptable to the patients, providing good cosmetic results and thereby patient satisfaction. Thus, mini-preauricular incision may be used as a convenient approach for open reduction and internal fixation of subcondylar fractures of the mandible.
